# RETRACTION: Effect of Various Repositioning Regimens on Pressure Wound Ulcer Occurrence in At‐Risk Adult Persons Without Existing Pressure Wound Ulcers: A Meta‐Analysis

**DOI:** 10.1111/iwj.70184

**Published:** 2025-01-12

## Abstract

**RETRACTION**: ZengM. , LiY. , HuJ. , PengM. , HuY. , and ZhouC. , “Effect of Various Repositioning Regimens on Pressure Wound Ulcer Occurrence in At‐Risk Adult Persons Without Existing Pressure Wound Ulcers: A Meta‐Analysis,” International Wound Journal 20, no. 9 (2023): 3776–3785, 10.1111/iwj.14277.37381159 PMC10588354

The above article, published online on 28 June 2023, in Wiley Online Library (http://onlinelibrary.wiley.com/), has been retracted by agreement between the journal Editor in Chief, Professor Keith Harding; and John Wiley & Sons Ltd. Following an investigation by the publisher, all parties have concluded that this article was accepted solely on the basis of a compromised peer review process. The editors have therefore decided to retract the article. The authors did not respond to our notice regarding the retraction.

**RETRACTION**: M. Zeng , Y. Li , J. Hu , M. Peng , Y. Hu , and C. Zhou , “Effect of Various Repositioning Regimens on Pressure Wound Ulcer Occurrence in At‐Risk Adult Persons Without Existing Pressure Wound Ulcers: A Meta‐Analysis,” International Wound Journal 20, no. 9 (2023): 3776–3785, 10.1111/iwj.14277.37381159 PMC10588354

The above article, published online on 28 June 2023, in Wiley Online Library (http://onlinelibrary.wiley.com/), has been retracted by agreement between the journal Editor in Chief, Professor Keith Harding; and John Wiley & Sons Ltd. Following an investigation by the publisher, all parties have concluded that this article was accepted solely on the basis of a compromised peer review process. The editors have therefore decided to retract the article. The authors did not respond to our notice regarding the retraction.

